# Maximizing the effectiveness of national commitments to protected area expansion for conserving biodiversity and ecosystem carbon under climate change

**DOI:** 10.1111/gcb.15645

**Published:** 2021-05-10

**Authors:** Carlos Carroll, Justina C. Ray

**Affiliations:** ^1^ Klamath Center for Conservation Research Orleans CA USA; ^2^ Wildlife Conservation Society Canada Toronto ON Canada

**Keywords:** climate change adaptation, climate change mitigation, climate velocity, connectivity, conservation planning, protected areas, refugia

## Abstract

Global commitments to protected area expansion should prioritize opportunities to protect climate refugia and ecosystems which store high levels of irrecoverable carbon, as key components of an effective response to biodiversity loss and climate change. The United States and Canada are responsible for one‐sixth of global greenhouse gas emissions but hold extensive natural ecosystems that store globally significant above‐ and below‐ground carbon. Canada has initiated a process of protected area network expansion in concert with efforts at reconciliation with Indigenous Peoples, and acknowledged nature‐based solutions as a key aspect of climate change mitigation. The US, although not a party to global biodiversity conventions, has recently committed to protecting 30% of its extent by 2030 and achieving the UNFCCC Paris Agreement's mitigation targets. The opportunities afforded by these dual biodiversity conservation and climate commitments require coordinated national and regional policies to ensure that new protected areas maximize biodiversity‐focused adaptation and nature‐based mitigation opportunities. We address how global commitments can best inform national policy initiatives which build on existing agency mandates for regional planning and species conservation. Previous analyses of global conservation priorities under climate change have been tenuously linked to policy contexts of individual nations and have lacked information on refugia due to limitations of globally available datasets. Comparison and synthesis of predictions from a range of recently developed refugia metrics allow such data to inform planning despite substantial uncertainty arising from contrasting model assumptions and inputs. A case study for endangered species planning for old‐forest‐associated species in the US Pacific Northwest demonstrates how regional planning can be nested hierarchically within national biodiversity‐focused adaptation and nature‐based mitigation strategies which integrate refugia, connectivity, and ecosystem carbon metrics to holistically evaluate the role of different land designations and where carbon mitigation and protection of biodiversity's resilience to climate change can be aligned.

## Graphical abstract

Global and national commitments to protected area expansion should prioritize protection of climate refugia and ecosystems which store high levels of irrecoverable carbon, as key components of effective response to biodiversity loss and climate change. The US and Canada have committed to protecting 30% of their respective lands and waters by 2030 and achieving ambitious carbon mitigation targets. We describe how these countries' regional conservation efforts can be coordinated within national strategies which use recently‐developed metrics to identify where carbon mitigation and protection of biodiversity's resilience to climate change can be aligned.

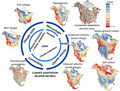

## 1 INTRODUCTION

The accelerating pace of biodiversity loss and climate change have prompted increasing calls for transformative change, including expansion of the global protected area network (Grumbine & Xu, 2021; IPBES, 2019). Although substantial progress has been made toward increasing protected area coverage globally, new areas have often not been sited with regard for their efficacy in safeguarding biodiversity or resilience to climate change (Díaz et al., 2019). Leaders of both the United States and Canada have joined those of other nations in endorsing global calls to protect at least 30% of their respective nations (ECCC, 2020a; White House, 2021). However, translating such commitments into national and subnational policies that maximize the role of new protected areas in addressing both climate change and biodiversity loss is challenging (Arneth et al., 2020). Here we explore how expansion of the protected area network within Canada and the United States can more effectively address climate‐change‐related threats to biodiversity by protecting climate refugia and areas with high levels of carbon stored within intact (i.e., high ecological integrity) ecosystems.

**FIGURE 1:**
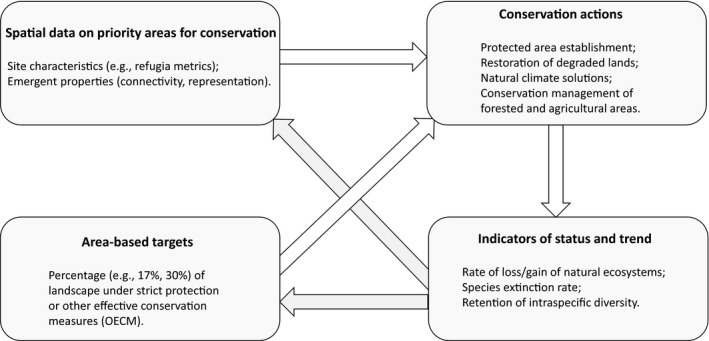
Diagram depicting the relationship between targets, indicators, spatial data, and conservation actions described in this review. Conservation actions are informed by area‐based targets and data on the location of priority areas such as refugia and areas of irrecoverable ecosystem carbon. Monitoring of the influence of conservation actions on biodiversity trends then informs revision of area‐based targets and spatial metrics

Protected areas are an essential component of a broader suite of strategies for sustainable coexistence between humans and the natural world, which includes restoration of degraded lands and conservation management of agricultural landscapes (Figure 1) (Strassburg et al., 2020). Protected area networks designed without information on how climate‐change‐related threats vary across the landscape may poorly protect biodiversity into the future (Carroll & Noss, 2020; Carroll et al., 2017; Stralberg, Arseneault, et al., 2020). Priorities based on current patterns of biodiversity may fail to capture important locations if, for example, species shifts toward cooler climates increase the conservation value of high elevation areas (Carroll et al., 2010; Holsinger et al., 2019; Rowland et al., 2016).

Certain areas play a disproportionate role in allowing landscapes to retain their capacity to support native species and ecosystems in the face of climate change (the focus of climate adaptation planning in the biodiversity context used here). Climate refugia (areas buffered from climatic shifts where organisms can persist permanently or temporarily) represent “slow lanes for climate change” which temporarily limits biodiversity loss while nations work to reduce their greenhouse gas emissions (Morelli et al., 2020). Refugia are created by a variety of factors ranging from local topography to broad‐scale climate gradients (Carroll et al., 2017; Morelli et al., 2020; Stralberg et al., 2018). Climate connectivity areas or corridors also play a key role by facilitating dispersal to newly climatically suitable habitat, which allows such habitat to augment the role of refugia (Carroll et al., 2018; Parks et al., 2020). Although climate refugia are not the only landscape element important to biodiversity conservation, they are an essential conservation feature which is increasingly feasible to identify from broad‐scale spatial data (Carroll et al., 2017; Stralberg, Carroll, et al., 2020).

Increased attention is also needed to the essential role of natural ecosystems in ameliorating climate change through their capacity to remove carbon dioxide from the atmosphere and offset direct emissions from other sectors (Goldstein et al., 2020; Morecroft et al., 2019). Proactive protection of above‐ and below‐ground carbon within intact ecosystems provides an important mitigation pathway distinct from that offered by improved intensive management or restoration of agricultural, grassland, and forested landscapes, such as widely publicized tree planting initiatives (Fargione et al., 2018; Griscom et al., 2017; Law et al., 2018; Seddon et al., 2021). An approach that focuses on avoided conversion of natural areas at meaningful scales is especially important in landscapes such as peatlands and old forests that hold large quantities of ecosystem carbon that, if lost due to disturbance, would be irrecoverable within a timescale meaningful to addressing climate change (Beaulne et al., 2021; Goldstein et al., 2020; Law et al., 2018). The large amount of land required, the convergence of biodiversity and climate change agendas (Roberts et al., 2020), and feedbacks between biodiversity loss and climate change (Arneth et al., 2020) should compel planners to seek to holistically align objectives for new protected areas that maximize co‐benefits (Díaz et al., 2020).

Previous analyses concerning global conservation priorities under climate change (Dinerstein et al., 2019, 2020; Jung et al., 2020; Maxwell et al., 2020; Yang et al., 2020) have largely lacked information on refugia locations due to limitations of globally available datasets, and have been only tenuously linked to the policy contexts of individual nations and regions. In this essay, we provide recommendations that can inform commitments for protected area network expansion at various scales so that they encompass areas that contribute to achieving climate and biodiversity targets. We first describe how existing global conservation targets address climate adaptation and mitigation goals, and how these can in turn best inform national and subnational protected area strategies. We evaluate high‐level policy opportunities for and barriers to achieving adaptation and mitigation goals at national and regional scales. We consider whether available data on refugia locations are adequate for guiding protected area establishment, and to what extent these locations overlap with priority areas for conserving ecosystem carbon. Finally, we use a case study to demonstrate how regional biodiversity‐focused adaptation and nature‐based mitigation strategies can be nested within national analyses of the role of different land ownerships and designations.

## 2 THE GLOBAL CONTEXT: PROTECTED AREA AND MITIGATION TARGETS IN INTERNATIONAL TREATIES

The first global commitment to biodiversity conservation was enshrined in 2010–2020 targets developed by the Conference of the Parties (COP) to the Convention on Biological Diversity (CBD) (CBD, 2010). The CBD has been ratified by 193 UN member states with the notable exception of the US. Although the CBD is a legally binding multilateral instrument, in practice the CBD's goals and targets advance conservation outcomes at the national level by stimulating ambition via pressure from civil society organizations, the scientific community, and peer policymakers in other signatory nations. Even within a nation such as the US which is not a party to the CBD, global targets indirectly influence policy proposals generated by policymakers and NGOs (White House, 2021).

The CBD's overarching goal is to reduce the rate of biodiversity loss and safeguard nature's contributions to people in an equitable manner (Díaz et al., 2020). The agreement to be finalized at the CBD's forthcoming meeting (COP15), termed the Global Biodiversity Framework (GBF), will include targets and indicators designed to comprehensively reflect the status of global biodiversity (CBD, 2021). The diversity of life is commonly recognized as being expressed at several scales: diversity among ecosystems, among species, and genetic diversity within species. Recent proposals have suggested that the GBF include targets for reducing the rate of loss and eventually halting loss of diversity at each of the three scales of biodiversity (Díaz et al., 2020). For example, proposed targets related to biodiversity status and trend include no net loss of intact ecosystems, reduction in species extinction rates, and retention of 90% of existing genetic diversity within species (Figure 1) (Díaz et al., 2020; Laikre et al., 2020; Watson et al., 2020).

The most widely publicized biodiversity‐related commitment adopted by the CBD involves area‐based targets (Figure 1). These include a 2010 commitment to protect 17% of terrestrial areas in ecologically representative and connected networks under strict protection or “other effective area‐based conservation measures” (OECM) (CBD, 2010; Jonas et al., 2014). This target may be raised to 30% at the CBD's forthcoming Conference of the Parties (CBD, 2021). Approximately 12% of the terrestrial US and Canada is currently strictly protected, but no standardized estimates are available for the extent of OECM lands (ECCC, 2020d; USGS, 2018).

The selection of targets and indicators included within the GBF, although informed by science, is ultimately a political process with negotiated outcomes. The goals of the framework include both comprehensively tracking the status of biodiversity at all levels and communicating this status to a wide audience of policymakers and the public. There is no single index of the status of biodiversity akin to the IPCC's focus on the magnitude of global mean temperature shifts. Area‐based targets such as 17% have been adopted by the CBD in part because they are easily communicated and measured, and have been linked conceptually and empirically to ecologically based targets such as reduction in species extinctions (Bhola et al., 2021; Noss et al., 2012). In fact, several recent publications have proposed that the 30% target is likely insufficient, particularly in geographies with more intensive human land use (Allan et al., 2019; Dinerstein et al., 2019; Noss et al., 2012).

Because biodiversity and the threats it faces are distributed unevenly across the landscape, the location of newly protected and conserved areas helps determine whether achieving an area‐based target results in effective progress toward the overarching goal of halting biodiversity loss, and secondary goals such as reduced species extinction rates (Díaz et al., 2020). The GBF specifies that monitoring of progress toward area‐based targets should also evaluate the spatial importance of new protected areas, in terms of both site characteristics (e.g., does the protected area capture hotspots of species richness?) and emergent properties of the protected area system as a whole (e.g., is the resulting network representative and connected?) (Figure 1).

Both area‐based targets and spatial data on priority areas (e.g., refugia) can inform conservation actions such as protected area establishment (Figure 1). With sufficient resources and effective management, newly protected areas will in theory contribute to improvements in status and trend indicators and achievement of associated targets (e.g., no net loss of ecosystems). However, due to the complexity of ecological systems, this connection may be indirect and subject to substantial time lags (Figure 1). Area‐based targets and spatial data on conservation priorities are themselves based on incomplete information and should be revisited and iteratively refined based on information derived from monitoring of biodiversity status and trends (Figure 1).

Global systems such as the Key Biodiversity Area (KBA) standard are intended in part to support site selection and make increases in protected area coverage more effective in improving biodiversity status and trends (Smith et al., 2019). Similarly, Dinerstein et al. (2019, 2020) described a global network of priority areas that would fulfill the GBF's 30% area‐based targets via protection of sites with high species diversity which as a whole would represent all ecoregions and form a well‐connected network of sites.

In 2015, signatories to the United Nations Framework Convention on Climate Change (UNFCCC), including Canada and the US, endorsed the Paris Agreement on reduction and mitigation of anthropogenic greenhouse gas emissions. A substantial component of the Agreement's goal for reduction and mitigation of emissions is associated with changes in land management and land use practices (IPCC, 2019; UNFCCC, 2015). Protected area designation forms one element of a broader strategy that must include improved management of the majority of lands and oceans of the planet managed by humans (Ellis, 2019; Locke et al., 2019).

Just as biodiversity loss and carbon emissions are considered in different global agreements, national commitments rarely address the two threats in a coordinated manner. In contrast, the 2015 United Nations Sustainable Development Goals contains both biodiversity and climate goals, and recognizes that protecting natural areas for adaptation and mitigation can also yield collateral ecosystem services by ensuring clean water supplies and buffering developed landscapes from extreme weather events such as flooding (Díaz et al., 2019, 2020; Zeng et al., 2020). The draft post‐2020 GBF also includes targets for such ecosystem services (CBD, 2021).

The GBF is novel in that it creates specific targets that provide opportunities to potentially align UNFCCC mitigation commitments and CBD biodiversity‐related goals (Arneth et al., 2020). The GBF's Target 7 proposes that by 2030, “nature‐based solutions and ecosystems‐based approaches” will provide increased contributions to climate change mitigation and adaption (CBD, 2021). Achievement of this target would be monitored in part via trends in carbon stocks in different ecosystems, and the contribution of natural ecosystems to climate change adaptation. The goal of greater coordination of climate adaptation and mitigation actions in the context of biodiversity conservation has received support from the High Ambition Coalition, an alliance of ~50 countries (including Canada) within the UNFCCC, and from the 65 CBD member states that are signatories to the 2020 Pledge for Nature calling for a unified “One Health” approach to addressing biodiversity, climate, and environmental issues (UNEP, 2020).

## 3 HOW CAN NATIONAL COMMITMENTS TO PROTECTED AREA EXPANSION EFFECTIVELY ADDRESS BIODIVERSITY LOSS AND CLIMATE CHANGE?

Policymakers encounter several challenges when they attempt to incorporate climate adaptation and mitigation priorities into protected area expansion efforts. Firstly, limited guidance is available on how global targets and indicators can best stimulate and inform national and subnational policy and regional conservation plans. Secondly, our knowledge on how to identify areas important for biodiversity, let alone climate refugia and corridors, and hence track progress toward their combined protection, is imperfect and constantly evolving. Lastly, priority areas for adaptation and mitigation may occur in different regions or require different management regimes, raising questions about how best to coordinate the two goals with one another and with other considerations related to biodiversity and ecosystem services.

Because area‐based targets such as 30% are simple and relatively easy to track, they have received most attention in national commitments (Díaz et al., 2019; Maxwell et al., 2020; Visconti et al., 2019). However, an exclusive focus on area‐based targets can lead to a mismatch between the location of new protected areas and regions of high value to biodiversity, adaptation, and mitigation (Carrasco et al., 2021; Maxwell et al., 2020; Morecroft et al., 2019; Visconti et al., 2019). Because many factors are considered in siting protected areas, recent studies have found that new conservation areas established in response to global commitments have had limited success in capturing areas of highest importance to current biodiversity (Maxwell et al., 2020) or climate refugia (Carrasco et al., 2021). Conversely, unlike in the case of area‐based targets, tracking progress toward adaptation and mitigation goals (e.g., COP15 Target 7) is difficult in part because of a scarcity of well‐developed metrics for measuring achievement.

Although previous publications concerning global conservation priorities under climate change provide essential context, they have been limited by globally available datasets (Dinerstein et al., 2019, 2020; Jung et al., 2020; Maxwell et al., 2020; Yang et al., 2020). The databases used in conservation planning processes (including global prioritizations) typically include information on current distribution of globally threatened taxa as proxies for biodiversity; they rarely include spatial data on climate refugia or climate connectivity areas, or address the additional uncertainty that arises from projecting species' response to climate change.

**FIGURE 2:**
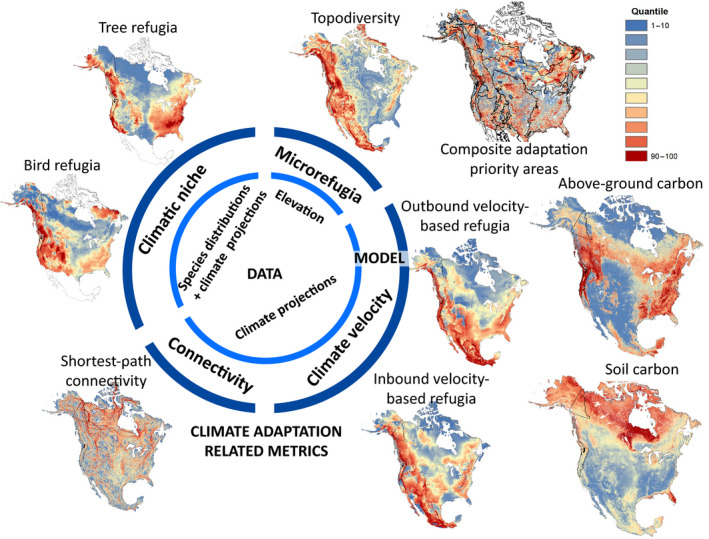
Range of patterns shown by nine metrics relevant to biodiversity‐focused climate adaptation and nature‐based mitigation in North America, categorized by type of input data and underlying model. All data were rescaled to equal‐area quantiles ranging from low (blue) to high (red) for comparability. See Supporting Information S1 for data sources and definitions of metrics

Priority setting at national or subnational extents can take advantage of a wider range of data on climate refugia and other adaptation targets than is available at the global extent (Figure 2). Nonetheless such data are more limited than information on current biodiversity patterns, in part because they are based on recently developed metrics (Table 1). Whereas the ecological responses of species to climate change are complex, the metrics used to map climate refugia are based on simplified representations of these dynamics. In this essay, we compare several major categories of metrics commonly used to identify refugia, climate corridors, and ecosystem carbon (Table 1, Figure 2; see Text S1 for definition of metrics).

**TABLE 1:** Refugia‐related metrics categorized by scale of biodiversity and type of metric. Although not all of the referenced publications specifically mention refugia, the methods listed can be used to identify refugia as areas where the current and projected future distribution of a feature overlap or are in proximity. See Supporting Information for further description of metrics

Scale of biodiversity	Type of metric		
	Species‐based (fine‐filter)	Non‐species‐based (coarse‐filter)	Mechanistic (e.g., focal species)
Ecosystems/communities	Bioclimatic Ecosystem Resilience Index^1^	Climatic niche models of ecoregions, vegetation types^2^	Dynamic global vegetation models of biome shifts^3^
Species	Refugia based on species' climatic niche models^4^	Not applicable	Refugia and colonizable areas based on species dispersal models^5^
Intraspecific/genetic	Climatic partitions of species' ranges^6^	Climate‐type‐based refugia and corridors^7^	Spatially explicit population genetic models^8^

References: 1. Ferrier et al. (2020); 2. Watson et al. (2013); 3. Eigenbrod et al. (2015), Gonzalez et al. (2010); 4. Stralberg, et al. (2018), Warren et al. (2018); 5. Miller and McGill (2018), Phillips et al. (2008); 6. Hanson et al. (2020), Rochat et al. (2021); 7. Carroll et al. (2017), Carroll et al. (2018); 8. Pierson et al. (2015).

## 4 CHALLENGES IN IDENTIFYING ADAPTATION PRIORITY AREAS AND TRACKING PROGRESS TOWARD THEIR PROTECTION

An ideal framework for tracking and protecting biodiversity under climate change would include targets and indicators which are coherently linked across scales of biodiversity. The GBF's three‐level framework (ecosystems, species, and genes) can be complemented by another categorization of ecological indicators, the three‐track framework for systematic conservation planning (Noss et al., 2002; Noss & Cooperrider, 1994). This latter approach suggests that protected area networks be designed to adequately represent both coarse‐filter or non‐species‐specific landscape elements (e.g., vegetation types) and fine‐filter or species‐specific habitat features, and also fulfill requirements (such as connectivity) necessary for persistence of key ecosystem processes and the population viability of focal species such as large carnivores. The three‐track framework encompasses indicators of biodiversity composition (the coarse‐ and fine‐filter elements correspond to composition at the ecosystem and species scales, respectively), as well as function (focal species viability). As Díaz et al. (2020) (p. S16) stated, “Ecosystem integrity, currently defined to include functional, compositional, and structural/spatial components, is more elusive to monitor than ecosystem area, but no less crucial for the long‐term continuity of ecosystem functioning”.

We can integrate these two indicator frameworks to provide a comprehensive categorization of the varied approaches to identifying climate refugia (Table 1). At each of the three scales of biodiversity, practitioners can integrate information from metrics which do or do not incorporate species‐specific data, as well as metrics based on more complex process‐based or mechanistic models if available. Since all of the metrics shown in Table 1 have their strengths and weaknesses, comparison of spatial priorities suggested by multiple metrics is informative.

Practitioners identifying refugia and other priority areas at the broadest scale of biodiversity, that of ecosystems and communities, can apply information from each of these three categories (Table 1). The most common approach at the level of biomes uses process‐based models such as dynamic global vegetation models (DGVM), which simulate shifts in potential vegetation in response to climate (Eigenbrod et al., 2015; Gonzalez et al., 2010). Finer‐scale spatial units in this global ecosystem classification hierarchy, such as ecoregions, have been identified based on biogeographic and environmental discontinuities, and can be effective tools for representing current patterns of biodiversity at a global scale (Smith et al., 2018). Because process‐based models such as DVGM have not been applied to simulate shifts in such finer‐scale ecological types, their relative resilience and vulnerability to climate change is assessed using statistical models which identify a climatic niche for each ecoregion and project the niche forward under future climates (Watson et al., 2013) (Table 1). Although such ecoregion models do not directly consider species‐specific data, researchers have recently developed a metric termed the Bioclimatic Ecosystem Resilience Index that relates data on spatial turnover in species composition to existing and projected future environmental conditions (Ferrier et al., 2020).

At the next scale of biodiversity, that of species, a wide variety of approaches is available to identify priority areas under climate change. The draft GBF proposes that results from species‐specific climatic niche models for a large number of taxa be aggregated and used to create global maps of the relative vulnerability and resilience of biodiversity under climate change (Warren et al., 2018). In place of such statistical niche models, a variety of more complex mechanistic models of species range shifts have been developed. Some incorporate simple dispersal kernels while others are full spatially explicit population models (Heinrichs et al., 2019; Miller & McGill, 2018; Phillips et al., 2008).

Recent reviews have proposed that the GBF also include a focus on intraspecific biodiversity, for example, via a commitment to conserve 90% of existing genetic diversity (Díaz et al., 2020; Laikre et al., 2020). For species for which there are sufficient data, genetic viability under changing climates can be projected using a mechanistic simulation model such as a spatially explicit population model (Pierson et al., 2015). Alternately, statistical models associating distinct genotypes with specific environments can be projected to future climates to identify genotype‐specific refugia (Rochat et al., 2021). Where genetic data are lacking, climatic subdivisions of a species' range can serve as surrogates for intraspecific ecotypes (Hanson et al., 2020). Planners can work to preserve adaptive capacity under climate change by conserving populations in all significant subunits of a species environmental niche (Carroll et al., 2020).

Coarse‐filter or non‐species‐specific approaches to identifying refugia for locally adapted populations often make use of climate velocity metrics (Carroll et al., 2015; Hamann et al., 2015). Multivariate climate space is divided into thousands of unique types, and distance between each climate type's current and projected future locations is measured. Refugia where a climate type's current and projected future locations overlap or are in close proximity allow locally adapted populations to remain within their suitable climate tolerances as climate changes (Carroll et al., 2015). Various forms of climate‐velocity‐based metrics (e.g., outbound vs. inbound; Kling et al. (2020)) identify different types of coarse‐filter refugia (Supporting Information S1).

All the approaches described above use future climate projections, and are consequently limited by the spatial resolution of those projections to identifying macrorefugia (areas where broad‐scale climate is relatively stable and suitable for persistence). Microrefugia (small areas with locally favorable environments within otherwise unsuitable climates) may be important to persistence of species with modest area requirements under climate change (Dobrowski, 2011). Because microrefugia are often created by terrain‐related factors, topographic diversity (topodiversity) data are useful for identifying areas where a heterogeneous physical environment (e.g., steep elevation gradients or diverse aspects) increases the likelihood that species will be able to find nearby suitable habitat as climate changes (Carroll et al., 2017).

## 5 EMERGENT CHARACTERISTICS OF THE PROTECTED AREA NETWORK AS A WHOLE

Because the warming effects of carbon emissions are global, locating priority areas for protection in those ecosystems which hold and sequester the most carbon maximizes protection of the global land sink (Goldstein et al., 2020). This goal may contrast with biodiversity conservation strategies which, in addition to locating particular areas important for supporting requirements of individual species, require distributing such areas widely in order to maximize biodiversity representation within protected areas (Margules & Pressey, 2000; Watson et al., 2011). The GBF monitoring framework includes the goal that protected area networks be representative, as measured by indicators based on the distribution of ecosystems and species (Faith et al., 2008).

Recent reviews have proposed stratifying or tracking achievement of global area‐based targets by ecoregion (Dinerstein et al., 2019) or by biome, a higher‐level aggregation of globally defined ecoregions (Stralberg, Carroll, et al., 2020). Representation of such spatial units is one of the few measures that can be reliably tracked with currently available data, although planners must choose between several available global delineations of ecoregions. However, to the extent that such ecoregional units are defined by climate, shifts in climate over coming decades may reduce their utility as strata for tracking representation of biodiversity.

A related issue involves how national responsibilities for achieving a target are distributed over subnational jurisdictions (provinces or states). Should opportunities for new protected areas be foregone in those parts of a nation which have already achieved the percentage target? This issue is especially important in nations such as the US where federally managed lands (and hence most opportunities for new protected areas) are concentrated in a few regions (ECCC, 2020d; USGS, 2018).

An additional potential concern is how actions at smaller extents will be coordinated to achieve broader goals such as facilitating connectivity. The goal of increasing connectivity between existing protected areas must be balanced with efforts to protect ecosystems in regions which are currently underrepresented in the protected areas network. Novel elements of connectivity also must be considered, such as the climate corridors which facilitate species' range shifts under climate change (Carroll et al., 2018). Climate corridors are areas that form the best route between current and future locations of specific climate types. Because dispersing organisms may need to avoid hostile climates, these routes are often circuitous rather than the straight‐line paths (Dobrowski & Parks, 2016).

Models that predict climate refugia and corridors based on future climate projections (inner circle, Figure 2) are inherently uncertain due to variation between global climate models and policy uncertainty regarding future rates of greenhouse gas emissions (Belote et al., 2018). (Metrics shown here were calculated as anomalies from the current (1981–2010) projected temperature and precipitation to the 2071–2100 period, based on an ensemble mean of 15 representative CMIP5 AOGCMs for representative concentration pathway (RCP) 8.5; Wang et al., 2016). Such uncertainty in input data is compounded by that inherent in the assumptions of the climate velocity (Ordonez & Williams, 2013) and connectivity models themselves (outer circle, Figure 2). Metrics based on physical habitat data such as elevation (Figure 2) avoid uncertainty related to projecting future climate, but retain substantial uncertainty regarding their accuracy at representing the complex factors which generate topographically driven microrefugia (Carroll et al., 2017; Dobrowski, 2011; Lawler et al., 2015).

Climatic niche models, which predict refugia are based on correlations between species distributions and current climatic conditions, provide a useful approximation of potential shifts in species' distribution in response to climate change (Wiens et al., 2009). However, they assume that current species distributions are at equilibrium in respect to climate, and ignore that the effect of climate change on many species is mediated by sympatric species and ecosystem structural components. For example, in the US Pacific Northwest region discussed in the case study below, the effect of climate change on many species is initially limited by the ecological inertia and microclimates created by large old trees (Carroll, 2010; Carroll et al., 2010; Perry et al., 2011). Subsequent shifts may be abrupt as fire disturbance regimes amplified by climate trigger ecosystem transitions (Williams et al., 2020). Conservation of fire refugia (areas disturbed less frequently or less severely by wildfire due to topography or other factors) in such systems is an important element of climate adaptation strategies (Krawchuk et al., 2020; Meddens et al., 2018).

These types of model uncertainty are not unique to climate adaptation planning, being also encountered by conservation planners when they attempt to optimize placement of new protected areas for multiple aspects of biodiversity despite lacking information on the distribution and ecological requirements of a large proportion of species (Reside et al., 2018; Watson et al., 2011). Such uncertainty can be addressed via quantitative methods that down‐weight priority areas with uncertain conservation value (Kujala et al., 2013; Moilanen et al., 2006) or via guidelines for factoring uncertainty into site‐level management strategies (Belote et al., 2017). Comparative evaluation of priorities identified by different metrics in the context of the known strengths and limitations of the underlying models (Figures 4, 5, 6, 7) is often more informative than attempting to identify a single optimal metric, and incentivizes holistic management strategies which address all facets of biodiversity's resilience to climate change (Carroll & Noss, 2020; Díaz et al., 2020).

A major component of the GBF has involved development of metrics to track achievement of global biodiversity targets (CBD, 2021). However, many of the metrics we discuss here, such as climatic niche models, are challenging to develop as consistent global datasets at a resolution relevant to national or regional planning, due to computational challenges or limited input data. Rather than propose new adaptation‐related GBF indicators that could be used to track progress globally, or evaluating the sufficiency of particular area‐based targets, we focus here on demonstrating how national and regional datasets can be used to maximize the effectiveness with which area‐based global targets (e.g., 30%) advance adaptation and mitigation goals. To supplement ongoing work at the global level, national‐level efforts should include an inventory of relevant spatial data on refugia and connectivity, and guidance for its use by agencies in regional planning. Formal or informal learning networks, such as the Landscape Conservation Cooperatives described below, can play a key role in comparing and learning from processes in different regions.

## 6 CHALLENGES TO COORDINATING ADAPTATION AND MITIGATION

Some recent proposals envision that the 30% of the landscape devoted to biodiversity protection could be distinct from “climate stabilization areas” managed for conservation of ecosystem carbon (Dinerstein et al., 2019, 2020). However, given the extent of land considered in these proposals, it is important to co‐locate new protected areas based on both adaptation and mitigation goals where possible, while integrating these in a manner consistent with wider biodiversity and sustainable development goals (Morecroft et al., 2019). There are frequently commonalities between optimal management strategies for conservation of climate refugia and irrecoverable carbon as both benefit from maintaining intact ecosystems. For example, old trees in coastal forests of the US Pacific Northwest and other regions store substantial carbon while providing refugia for rare and endemic species in part by moderating local microclimate (Spies et al., 2019). In boreal North America (e.g., the Hudson Bay Lowlands), protecting carbon‐rich boreal peatlands also safeguards key climate refugia due to the moderating microclimatic influence of mesic areas (Stralberg, Arseneault, et al., 2020).

**FIGURE 3:**
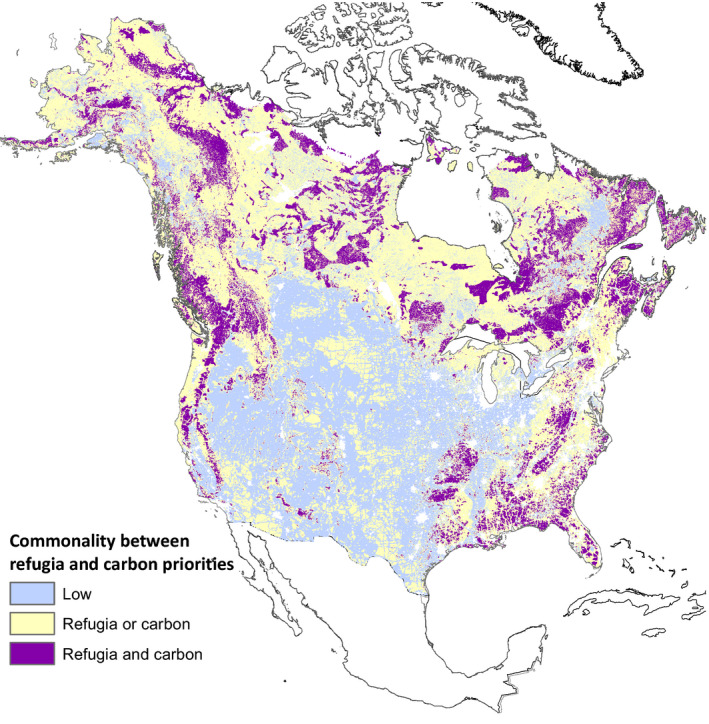
Overlap between the 30% of area with highest value for (1) climate adaptation, as identified by Stralberg, Carroll, et al. (2020) from a composite prioritization based on 5 climate refugia and connectivity metrics, and (2) above‐ground carbon (Spawn et al., 2020) and/or soil carbon levels (Hengl et al., 2017)

In reality, commonalities between ecosystem carbon and either current biodiversity or refugia are often weak, especially in arid environments (Di Marco et al., 2018). At the extent of the United States and Canada as a whole, we found that climate adaptation priority areas identified by Stralberg, Carroll, et al. (2020) are effectively uncorrelated with both above‐ground carbon (Spawn et al., 2020) and soil carbon (Hengl et al., 2017) (rank correlation on 0.016 and −0.031, respectively). We acknowledge that the metrics used here to measure carbon stocks do not fully represent the diversity of metrics that can be used to assess the carbon mitigation potential from conservation investments (e.g., Goldstein et al., 2020). The 16.0% of the two nations which falls in the top three deciles (30%) of value for both adaptation and ecosystem carbon (Figure 3) is divided between boreal regions with high soil carbon and high‐biomass mesic forests of the Pacific coast and eastern North America. While this particular representation of joint adaptation and mitigation priorities is incomplete (e.g., it ignores restoration opportunities in grassland ecosystems; Strassburg et al., 2020), it demonstrates that areas of joint adaptation and mitigation potential are limited and may be priorities for protection.

Additionally, there may be tradeoffs between management for adaptation and mitigation, for example, in those ecosystems where suppression of fire disturbance provides short‐term increases in carbon retention but negatively affects native species (Perry et al., 2011). The complexities of carbon management in such ecosystems will require planners to consider a range of management strategies, including more intensive management than tends to be associated with strictly protected areas (Belote et al., 2017).

Additionality and permanence are core aspects of quality assurance of climate change mitigation processes, particularly carbon offset projects (Federici et al., 2017). The imperative to consider both creates a potential mismatch between conservation of irrecoverable carbon within intact ecosystems and market‐based systems for tracking mitigation commitments. To receive payments for nature‐based mitigation via conservation of ecosystem carbon, managers must typically demonstrate that actions (e.g., protected area designation) result in avoidance of otherwise likely additional carbon loss (Federici et al., 2017). Pre‐existing biodiversity conservation commitments within newly protected areas can hamper the ability to demonstrate additionality of carbon conservation agreements. Protection of old forest from timber harvest in regions with infrequent natural disturbance provides measurable additionality (Buotte et al., 2020). In contrast, it is often difficult to establish additionality resulting from protection of below‐ground carbon (e.g., boreal peatland complexes) unless the area is under immediate threat from mining or other ground‐disturbing activities.

Due to the stochastic nature of large disturbance events, it is difficult to predict the risk of loss of above‐ground carbon to fire in many forest ecosystems (Anderegg et al., 2020). This makes it challenging to demonstrate permanence of claimed carbon benefits for any single protected area. Carbon markets currently account for unexpected carbon loss from fire by tapping a reserve of offset credits. The National Greenhouse Gas Inventory (NGHGI) framework developed under the Paris Agreement may offer a more appropriate method for tracking carbon benefits from protected area expansion while accounting for short‐term fluctuations due to disturbance (Federici et al., 2017).

## 7 PROTECTED AREA EXPANSION THROUGH RECONCILIATION‐BASED LAND USE PLANNING IN CANADA

Although the CBD's area‐based targets have helped propel a global expansion of protected area networks, the pace of expansion has varied widely among nations (Woodley et al., 2019). Canada and the United States, as an adopter and non‐adopter, respectively, of global biodiversity conventions, provide an illustrative contrast. The two nations are responsible for one‐sixth of global greenhouse gas emissions but also hold extensive areas where natural ecosystems store globally significant above‐ and below‐ground carbon (Coristine et al., 2019). The US is among the 17 “megadiverse” countries of the world (Pariona, 2018) and Canada contains the second highest remaining area of intact ecosystems globally (Coristine et al., 2019; Watson et al., 2018).

The Canadian federal government, as a signatory to both the CBD and the Paris Agreement, has made high‐level commitments to both the 17% and 30% protection targets and mitigation of carbon emissions (ECCC, 2019, 2020a, 2020b; MacKinnon et al., 2015; Zurba et al., 2019). Each CBD signatory nation is required to develop a national biodiversity strategy and action plan. Canada's strategy, as revised in 2015, contains a commitment (termed Canada's Target 1) to conserve at least 17% of the terrestrial landscape by 2020 (ECCC, 2016). However, Canada faces significant barriers to achieving global biodiversity targets. Due to the constitutional division of jurisdiction between federal and provincial governments, the 10 provinces have exclusive jurisdiction over most public land, with only 4% of land being federally administered. Designation of new protected areas therefore requires collaboration with the relevant provincial government. Canada's three northern territories are at various stages of devolution of land management responsibilities from federal to regional governments (Zurba et al., 2019).

There is a rising imperative to advance biodiversity conservation in the context of reconciliation through Indigenous‐led conservation (Artelle et al., 2019; Zurba et al., 2019). The Canadian federal government has coordinated efforts to achieve Target 1 with a stated commitment to reconciliation with Indigenous Peoples under the United Nations Declaration on the Rights of Indigenous Peoples (UNDRIP) (Table 2). The Indigenous Circle of Experts was convened in 2017 to examine how Canada's conservation and reconciliation commitments could be coordinated (ICE, 2018). As of 2019, the Canadian government had granted a total of $175 million to support 27 proposals for Indigenous Protected and Conserved Areas (IPCA) across Canada (ECCC, 2019).

**TABLE 2:** Examples of potential policy pathways for incorporating biodiversity‐focused climate adaptation and nature‐based mitigation into protected area network expansion and land management. Asterisks indicate proposed policies. The US government has endorsed UNDRIP's goals but did not declare it legally binding

Authority	United States	Canada
International treaties	UN Framework Convention on Climate Change (UNFCCC) UN Declaration on the Rights of Indigenous Peoples (UNDRIP)	UNFCCC UNDRIP Convention on Biological Diversity (CBD) Sustainable Development Goals (SDG)
Legislative	Endangered Species Act National Forest Management Act Federal Land Policy and Management Act revisions* Wildlife Corridors Conservation Act* National Strategic Carbon Reserve*	Species At Risk Act (SARA) and equivalent provincial/territorial statutes Protected area statutes
Executive		
National processes	Forest Service Roadless Area Conservation Policy Landscape Conservation Cooperative network Presidential climate policy memos DOI climate adaptation policy	A Healthy Environment, A Healthy Economy
Regional processes	Northwest Forest Plan revision (NFP) Sage grouse conservation plan Land and Water Conservation Fund (LWCF) Fish and Wildlife Service (FWS) consultation process Forest Service regional planning FWS recovery planning Department of Defense land management planning National monument designation Conservation Reserve Program	Pathway to Canada Target 1 Land use planning Caribou recovery planning
Non‐federal	State endangered species acts State Wildlife Action Plans (SWAP) State land conservation funds Conservation easements Carbon market mechanisms	Provincial wildlife and endangered species management and recovery planning Conservation easements Carbon market mechanisms

**FIGURE 4:**
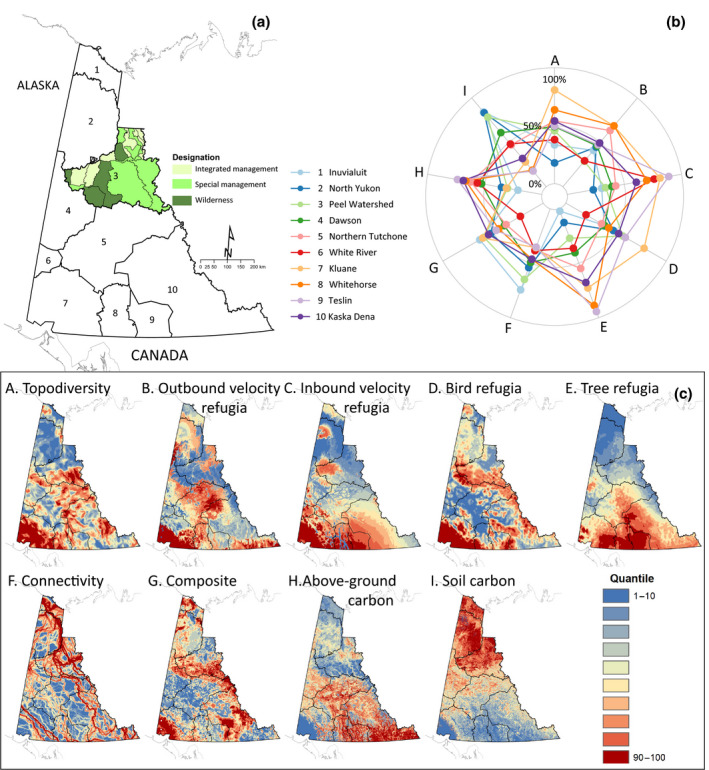
Values of nine metrics relevant to ecosystem‐based climate adaptation and mitigation within the Yukon Territory, Canada. (a) The Yukon is divided into 10 regions for land use planning purposes. A land use plan was ratified in 2019 for the Peel Watershed which places the majority of the watershed under conservation management. (b) The relative values of the nine metrics can be compared using a starplot. (c) Maps depicting the patterns of the nine metrics: (A) topodiversity, (B) refugia based on outbound climatic velocity, (C) refugia based on inbound climatic velocity, (D) bird species refugia, (E) tree species refugia, (F) shortest‐path connectivity, (G) composite priorities developed from a group of adaptation metrics by Stralberg, Carroll, et al. (2020), (H) aboveground carbon, and (I) soil carbon. See Supporting Information S1 for definition of metrics and data sources

An example of the nexus between reconciliation and protected area expansion is provided by IPCA in Yukon, Canada (Figure 4). A 2017 Supreme Court decision reaffirmed that under the 1993 Umbrella Final Agreement (UFA), no one government can unilaterally develop a land use plan without having brought all its interests and positions to the negotiating process in good faith. Comprehensive regional‐scale land use planning is mandated for each of 10 regions subject to the UFA within the Yukon Territory (Figure 4). For the three Yukon First Nations that did not sign the UFA, other recent court decisions ruled that they still have title and rights and that these cannot be negatively affected without consent. The alignment of the federal Target 1 process with regional land use planning provides a major opportunity for creation and expansion of IPCA. For example, the land use plan for the Peel Watershed, ratified in 2019, confers some level of protected status on 83% of the watershed (Figure 4a) (PWPC, 2019).

We illustrate how multiple refugia‐related metrics can be holistically assessed in planning processes using visual aids such as starplots. Starplots provide a compact way to integrate information from multiple metrics into a single diagram to produce a composite “fingerprint” representing the varying magnitudes of factors affecting climate adaptation and mitigation values (Figure 4b) (Carroll & Noss, 2020; Garcia et al., 2014). Starplots for the Yukon (Figure 4), which represent the average conservation value of a metric in each planning unit, scaled in comparison to the range of values shown across the entire region (e.g., the Yukon territory), provide an example of how the climate adaptation and mitigation metrics reviewed here can inform planning.

The starplot patterns (Figure 4b) suggest that ecosystems with highest soil carbon lie within the taiga and tundra regions in the northern Yukon, whereas areas with greatest value as climate refugia are found in areas of higher topographic relief in the central and southwest Yukon (Figure 4c). Areas important for climate connectivity (in the non‐species‐specific form considered here) are found throughout the region. Although the continental‐extent data we present here will be too coarse‐resolution for some planning processes, several efforts are completed or ongoing to develop climate‐adaptation‐related data at higher spatial resolution to help inform conservation planning co‐led by First Nations in the Yukon (e.g., BEACONS, 2017; Cooke, 2017; Stralberg, Arseneault, et al., 2020).

Canada's effort to reach protected area targets promises to achieve important additional societal benefits via its linkage to reconciliation processes (Artelle et al., 2019; Zurba et al., 2019). Such an inclusive planning model has broad relevance to other communities which have been historically unrepresented in land use decisions or negatively impacted by protected area expansion. Given that existing carbon market mechanisms are poorly suited for carbon conservation within intact ecosystems, First Nations are exploring new mechanisms for generating carbon‐related payments to help support local communities in lieu of revenue from extractive industries such as logging, oil and mining (Townsend et al., 2020). These mechanisms may also be applicable to other rural economies dependent on revenue from public lands.

While opportunities for Indigenous‐led conservation and conservation of public (Crown) land are significant in Canada, the fragmented nature of jurisdiction and the primacy of natural resource development for revenue create significant barriers to progress (Ray et al., 2021). Nevertheless, the globally significant carbon storehouses (including the second largest peatland complex on the planet, the Hudson Bay Lowlands) and sizeable areas characterized by high ecological integrity (Grantham et al., 2020) offer pressing reasons to actualize government commitments.

## 8 PROTECTION OF REFUGIA AND CARBON‐RICH ECOSYSTEMS VIA EXECUTIVE BRANCH ACTION IN THE UNITED STATES

The incoming US federal administration has committed to the target of 30% protection by 2030 (White House, 2021). Policymakers have called for focusing protection on lands which would aid in preventing extinction, stabilizing ecosystems, and sequestering carbon and greenhouse gas emissions. This “30x30” initiative offers an opportunity to advance both adaptation and mitigation goals as the US simultaneously recommits to the UNFCCC's Paris Agreement (Rosa & Malcom, 2020). The confirmation of the first Indigenous Secretary of the US Department of the Interior has placed increased focus on how tribal co‐management approaches can jointly address reconciliation and climate issues, and such approaches have been highlighted in recent executive orders (White House, 2021).

The separation of powers between the US executive and legislative branches frequently results in a divided government that presents barriers to ratification of international agreements and legislation related to climate mitigation and adaptation (Snape, 2009). In anticipation of such hurdles, the Paris Agreement was structured so that ratification by the legislative branch would not be required for US endorsement (UNFCCC, 2015). Although the advent in 2021 of a new US administration marks a significant shift toward a greater focus on climate mitigation and adaptation (White House, 2021), US federal initiatives may remain primarily limited to executive branch directives and opportunities within regional planning processes. Although unlike environmental statutes, executive rulemakings can be rescinded by a new administration, they have historically provided significant opportunities for expansion of the US protected area network.

**FIGURE 5:**
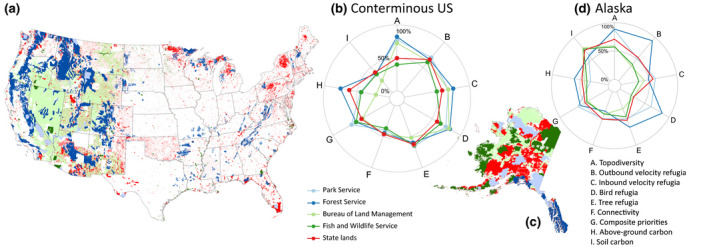
Major land management categories within (a) the conterminous United States and (c) Alaska, and (b, d) the relative values of nine ecosystem‐based climate adaptation and mitigation metrics within those categories. Colors for respective categories in the map match those used in the starplot. See Supporting Information S1 for definition of metrics and data sources

Unlike in Canada, lands over which the US federal government has direct authority constitute a substantial proportion (~28%) of the nation as a whole, and an even greater proportion of the 11 contiguous western states (46.4%; Figure 5a) and Alaska (61.3%; Figure 5c) (CRS, 2020). The Department of Interior's Bureau of Land Management, Fish and Wildlife Service, and National Park Service manage 10.8%, 3.9%, and 3.5% of the nation's land, respectively, while the Department of Agriculture's Forest Service manages 8.5% (CRS, 2020). Starplots (here created separately for the distinct contexts of contiguous US, Figure 5b and Alaska, Figure 5d) suggest contrasts in adaptation and mitigation values for lands managed by the various agencies, and also highlight the conservation value of lands managed by the US states.

**FIGURE 6:**
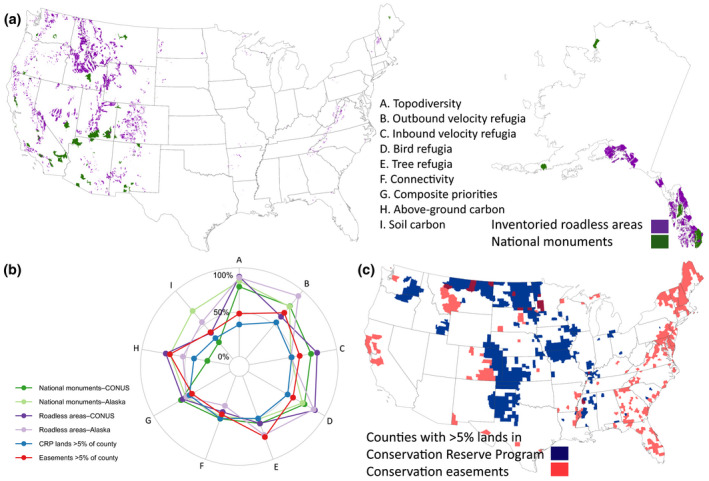
Four land management designations within the United States discussed in text as relevant to a national ecosystem‐based climate adaptation and mitigation strategy: (a) inventoried roadless areas on Forest Service lands and national monuments, (c) lands enrolled in the Conservation Reserve Program (CRP) and conservation easements. CRP lands and easements are summarized by county due to limited spatial resolution of the datasets, with data limited to the conterminous US as no Alaskan counties met the specified threshold. The relative values of nine metrics relevant to ecosystem‐based climate adaptation and mitigation are compared using a starplot (b). See Supporting Information S1 for definition of metrics and data sources

Although not directly informed by climate adaptation and mitigation goals, two types of executive branch actions have incidentally served to conserve climate refugia and carbon‐rich ecosystems. The 2009–2017 Obama administration added 21,000 km^2^ of terrestrial federal ownership to bring the network of terrestrial National Monuments designated by previous administrations under the Antiquities Act to approximately 76,000 km^2^ (CRS, 2019) (Figure 6a). The 2001 Roadless Area Conservation Policy ended most logging, road building, and mineral leasing on 235,000 km^2^ of undeveloped lands within the US national forest system (Talty et al., 2020) (Figure 6a).

These national monuments and roadless areas incidentally resulted in protection of conservation values that policymakers had not yet established as explicit goals, by safeguarding important climate refugia and high‐carbon ecosystems, especially within old‐growth temperate rainforests in southeastern Alaska and the US Pacific Northwest (Buotte et al., 2020; Talty et al., 2020) (Figure 6b). Adaptation and mitigation benefits from these existing initiatives should be assessed and integrated into any new national strategy to achieve the 30% protection target and national commitments under Paris Agreement. The current US administration has an opportunity to more purposefully prioritize protection of refugia and carbon‐rich ecosystems in agency policies or via designation as national monuments.

Comprehensive landscape planning for climate adaptation and mitigation must overcome barriers to coordination between multiple federal agencies with contrasting land management mandates (Mihm, 2000). A second requirement is sufficient political will. For example, pressure for expansion of renewable energy infrastructure on federal lands as part of federal efforts to reduce carbon emissions prompted development of the Desert Renewable Energy Conservation Plan (DRECP) to coordinate biodiversity conservation in in California's southeastern desert with expansion of solar, wind, and geothermal infrastructure (Kreitler et al., 2015).

## 9 CASE STUDY: OPPORTUNITIES FOR CONSERVATION OF CLIMATE REFUGIA AND CARBON UNDER THE NORTHWEST FOREST PLAN

The US planning processes with the greatest potential for expanding protection across multiple jurisdictions typically have occurred when the desire to avoid imminent listing of a species as endangered or threatened results in strong political support for overcoming bureaucratic barriers to coordination. For example, the 2015 planning process for conservation of the greater sage grouse (*Centrocercus urophasianus*) resulted in new protections on 67,000 km^2^ of habitat “strongholds” in 10 western states (Pidot, 2018).

**FIGURE 7:**
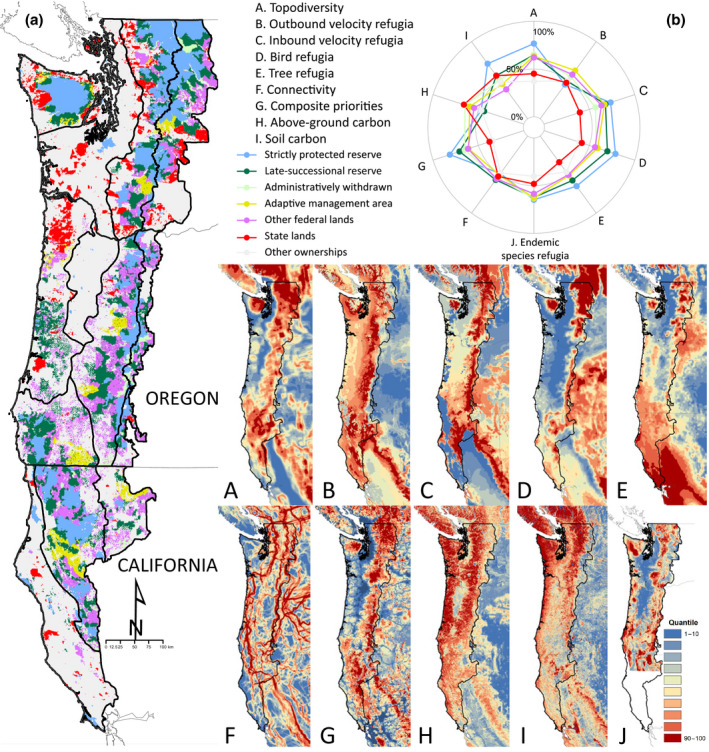
Values of 10 metrics relevant to ecosystem‐based climate adaptation and mitigation within the (a) region of the US Pacific Northwest where public land management is guided by the Northwest Forest Plan. The region is divided into the 12 physiographic provinces outlined in black for the purposes of stratifying conservation targets. Regional data on refugia for rare and endemic species developed by Carroll et al. (2010) are included in addition to the nine metrics portrayed in Figures 4, 5, 6. (b) The relative values of the ten metrics can be compared using a starplot. See Supporting Information S1 for definition of metrics and data source

The Northwest Forest Plan (NFP; Figure 7) provides another example which exhibits the two key requirements for coordinated multi‐agency conservation planning (Spies et al., 2019). Strong political impetus was generated when a judicial decision upended the status quo of timber management on federal lands, finding that it failed to conserve the northern spotted owl (*Strix occidentalis caurina*), whose declining population trends were related to the loss of older coniferous forest habitat (Noon & Blakesley, 2006). Barriers to cross‐jurisdiction planning were removed when President Clinton directed 10 federal agencies to coordinate planning to ensure viable populations of the owl and other old‐growth‐associated species over 79,000 km^2^ of the US Pacific states.

The NFP achieved this goal in part via designation of “late‐successional reserves” (Figure 7a) (Noon & Blakesley, 2006; Spies et al., 2019). Although not formally part of the protected area network, this new designation prioritizing protection of old forest and associated species falls under the CBD's OECM category. Strictly protected and OECM lands now total approximately 29% of the NFP region (Figure 7a). While the NFP has achieved conservation gains for the owl and other old‐growth‐associated species, recent reviews have called for upcoming plan revisions to incorporate new protections for climate refugia and ecosystem carbon, as well as other ecosystem services (Spies et al., 2019). The NFP offers an existing multi‐agency foundation for these updates which is not available in regions with more fragmented land management planning. Additional areas under conservation management may be necessary to achieve the NFP's conservation goals (Dunk et al., 2019), demonstrating that the 30% threshold is insufficient in some contexts (Figure 1).

The NFP region exemplifies the three challenges to adaptation and mitigation planning described previously. Planners must reconcile a diversity of data on the location of climate refugia. There are both commonalities and contrasts between adaptation priority areas as represented by refugia for rare and endemic old‐forest‐associated taxa (Carroll et al., 2010), songbird and tree species refugia (Stralberg et al., 2018), potential topographic microrefugia (Carroll et al., 2017) and climate connectivity areas (Carroll et al., 2018). Starplots of adaptation and mitigation values for the NFP region (Figure 7b) suggest that while protected and OECM lands administered by the Park Service and Forest Service will have the greatest role in protecting refugia, other categories such as state forestlands could also play a large role, for example, in protecting ecosystem carbon. Regional analyses such as this one can incorporate additional datasets (such as endemic species refugia; Carroll et al., 2010) which are not available at national or global extents.

The NFP region exhibits in microcosm the geographic contrast between adaptation and mitigation priority areas that is also evident at the continental extent. Priority areas for carbon protection are found predominantly in the northern coastal section of the NFP region, where carbon‐releasing fire disturbance is infrequent (Buotte et al., 2020). In contrast, the southern portion of in the NFP region hold greater importance for species diversity and species refugia (Figure S1) (Carroll et al., 2010). The original 1994 Northwest Forest Plan addressed representation issues by distributing late‐successional reserves across the region's physiographic provinces (outlined in Figure 7a). The NFP revision process would need to address whether adaptation and mitigation goals would be similar representative, or whether mitigation goals should primarily focus on wet coastal forests.

## 10 CONCLUSION: TOWARD BETTER INTEGRATION OF CLIMATE ADAPTATION AND MITIGATION GOALS INTO PROTECTED AREA NETWORK EXPANSION

A necessary foundation for effective biodiversity‐focused adaptation and nature‐based mitigation policy is a clear national strategy that is linked to agency mandates and regional planning processes. Such a strategy should address the issues identified above regarding the availability and appropriate use of spatial data, reconciling management for adaptation and mitigation, establishing and coordinating national and regional targets, and tracking progress toward target achievement.

Notwithstanding some encouraging progress to date sparked by Canada's Target 1 process, prioritizing new areas for protection faces numerous barriers in achieving biodiversity, climate adaptation, and mitigation goals. These range from data availability limitations to societal pressures, including economic opportunity costs of conservation. Achieving such outcomes will require that more explicit policy links be made between Canada's commitments under the CBD and the Paris Agreement, although some promising steps are being made with the recent issuance of a new climate plan that acknowledges the essential roles of nature and of Indigenous‐led conservation (ECCC, 2020c). Funding decisions so far have been substantially opportunistic (i.e., based on submitted proposals), rather than through proactive and systematic prioritization of conservation targets underrepresented within the existing protected area network. Although potential projects are widely distributed across Canada, most newly protected land has to date been located in a few regions (ECCC, 2020b). A process is needed that provides transparent evidence‐based evaluation of where protection of biodiversity, climate refugia, and carbon storehouses can be aligned. Moreover, even if both quality and quantity area‐based targets are met, biodiversity conservation will not be achieved without concerted attention to limiting the extent and intensity of development within unprotected areas (Ray et al., 2021).

Coordinating Canada's commitments under the CBD, the Paris Agreement, and the UN Sustainable Development Goals will require updating the existing National Biodiversity Strategy and targets (ECCC, 2016) and harmonizing the strategy with nature‐based carbon mitigation policy. Once the GBF is finalized, there will be an immediate need to broaden the focus beyond meeting area‐based targets to incorporate and track a greater range of GBF and other indicators. Including refugia and potentially mitigation priority areas along with biodiversity data such as the location of Key Biodiversity Areas (KBA Canada Coalition, 2021) would be one means of ensuring that progress in each CBD signatory nation is tracked under global accords.

The US, although it has no formal commitments under global biodiversity treaties, can support similar integration of adaptation and mitigation strategies via executive direction requiring multi‐agency coordination concerning protection of areas of high importance to biodiversity‐focused adaptation and nature‐based mitigation (Table 2). The 2009–2017 Obama administration promulgated policies which established climate adaptation and mitigation policy direction across multiple federal agencies which have significant jurisdiction over lands in the country (Office of the President, 2013). The US Department of Interior can build on these previous actions and incorporate a suite of measurable adaptation and mitigation targets into regional planning documents. Although the Bureau of Land Management already has the scope to mainstream climate adaptation and mitigation in planning (Pleune et al., 2020), proposed revision of the agency's enabling legislation (the Federal Land Policy and Management Act; Table 2) offers an opportunity to strengthen this mandate.

Efforts in the US as in Canada would benefit from development of a national biodiversity‐focused adaptation and nature‐based mitigation strategy which described a nested sequence of analyses that assess how regional actions can best contribute to achieving national targets and ensuring connectivity and complementarity among regions (Law et al., 2004). In this essay, we have provided examples of such complementary national (Figures 5 and 6) and regional‐scale (Figures 4 and 7) assessments. This proposal aligns with other recent mitigation‐focused proposals, such as for a National Strategic Carbon Reserve to prioritize retention of ecosystem carbon on federal lands, especially within moist forests in the US Pacific Northwest and Alaska (Dellasala et al., 2020).

Greater coordination of energy and natural resource production, mitigation, and climate adaptation policies in whole‐of‐government approaches, including re‐direction of subsidies for fossil fuel and natural resource development, is also essential (Ray et al., 2021). At a national level, such coordination could involve elimination of incentives for extractive energy production in climate refugia and carbon‐rich ecosystems, especially on public lands. Measures such as a fossil fuel leasing moratorium on public lands would limit both downstream effects of fossil fuel use and direct emissions during the extraction process (Eilperin & Grandoni, 2020). Growth‐inducing linear infrastructure, for example, pipelines and basin‐opening roads, also merits scrutiny outside of project‐specific environmental impact assessments (Johnson et al., 2020).

National biodiversity‐focused adaptation and nature‐based mitigation strategies should be integrated with other pathways for conservation management. In Canada, recovery planning mandated by the Species At Risk Act for widely distributed species of concern such as caribou (*Rangifer tarandus*) presents an opportunity for achieving co‐benefits from protection of refugia and ecosystem carbon (Wells et al., 2020). In the US, regional species conservation processes, such as those described above involving the sage grouse and northern spotted owl, should be broadened to consider adaptation and mitigation goals, and tiered to goals presented within the national strategy.

The Fish and Wildlife Service (FWS), the US agency responsible for conservation of terrestrial endangered and threatened species, not only manages land directly, but also consults with other land management agencies when the latter's actions may affect species of concern (Jeffers, 2008). Such “Section 7” consultations provide a potential pathway to advance coordinated protection of climate refugia across multiple jurisdictions. Designation of “critical habitat” for threatened and endangered species by FWS provides another pathway for refugia protection. However, recent policy changes (FR 85 82376) and case law (*Weyerhaeuser Co. v. U.S. Fish and Wildlife Service, 17‐71* (Nov. 27, 2018)), which prohibit FWS from designating critical habitat in an area where the species of concern is not currently present, are problematic in that they may prevent protection of areas which are newly suitable for a species due to shifting climates.

Private and non‐federal public lands harbor a significant proportion of North America's biodiversity (Rosa & Malcom, 2020) and climate refugia (Figure 6b). Federal governments can also use funding to incentivize participation by non‐federal landholders. Support of Indigenous‐led protected area proposals by Canada's Target 1 Challenge Fund provides an example of the potential impact of such programs (ECCC, 2020b). In the US, protection of refugia on non‐federal lands can be achieved via the Land and Water Conservation Fund, which provides matching funds to state and local authorities, and the Conservation Reserve Program (CRP), which pays private landowners to manage land for conservation (Szentandrasi et al., 1995). US counties with the highest proportion of CRP lands or conservation easements show high values for conserving climate connectivity and above‐ground carbon (Figure 6b). US states such as California which have endorsed area‐based protection targets and mitigation goals could also benefit from developing a coordinated adaptation and mitigation strategy that builds on existing State Wildlife Action Plans.

In 2009, the US Department of Interior established a network of 22 regional Landscape Conservation Cooperatives (LCC) in order to coordinate efforts by federal agencies, states, tribes, and non‐governmental organizations to address climate adaptation and other broad‐scale conservation issues (Jacobson & Robertson, 2012). However, LCC funding was terminated in 2019 by the subsequent federal administration, and although Canadian government staff and other experts participated in transboundary LLCs, funding from Canada was absent. Federal science agencies can play a key role in supporting biodiversity‐focused adaptation and nature‐based mitigation efforts by reviving and strengthening the network of Landscape Conservation Cooperatives and other “communities of practice” (Wenger, 1999) that bring together researchers and practitioners (e.g., staff from governmental agencies, First Nations, and NGOs).

Our policy recommendations are relevant to other nations which face similar challenges, in terms of both policy and information needs, in implementing and coordinating biodiversity‐focused adaptation and nature‐based mitigation. The examples we develop demonstrating how regional biodiversity‐focused adaptation and nature‐based mitigation strategies can be nested within national analyses of the role of different land ownerships and designations are applicable outside of North America. Given existing policy barriers and the strength of countervailing societal forces, it will be challenging to mainstream consideration of refugia, climate connectivity, and irrecoverable ecosystem carbon into planning processes (Ray et al., 2021). However, such ecosystem‐based strategies are a necessary component of any effective effort to address the interlinked crises of climate change and biodiversity loss (IPBES, 2019).

## CONFLICTS OF INTEREST

The authors declare no conflicts of interest.

## AUTHOR CONTRIBUTIONS

CC conceived the study; CC and JR wrote the paper.

## Supporting information

Supplementary Material

## Data Availability

All data presented in this study are openly available at adaptwest.databasin.org.
