# Supplementary material for: Maximizing the effectiveness of national commitments to protected area expansion for conserving biodiversity and ecosystem carbon under climate change
Source: Glob Chang Biol. 2021 May 10;27(15):3395–414. doi: 10.1111/gcb.15645 (PMC8360173; doi:10.1111/gcb.15645)
Supplement: Supplementary file 1 — Supplementary Material [file GCB-27-3395-s001.pdf]

Supporting information for “Maximizing the effectiveness of national commitments to protected area expansion for conserving biodiversity and ecosystem carbon under climate change”.

Carlos Carroll[1], Justina C. Ray[2].

1. Klamath Center for Conservation Research, Orleans, CA 95556 USA.

2. Wildlife Conservation Society Canada, Toronto, ON, M5S3A7 CANADA

SI Figure S1. Starplots for Northwest Forest Plan (NFP) region for 10 metrics relevant to ecosystem-based climate adaptation and mitigation stratified by management category (a), western physiographic provinces (b), and eastern physiographic provinces (c). Metrics A-J labeled as in main text figures.

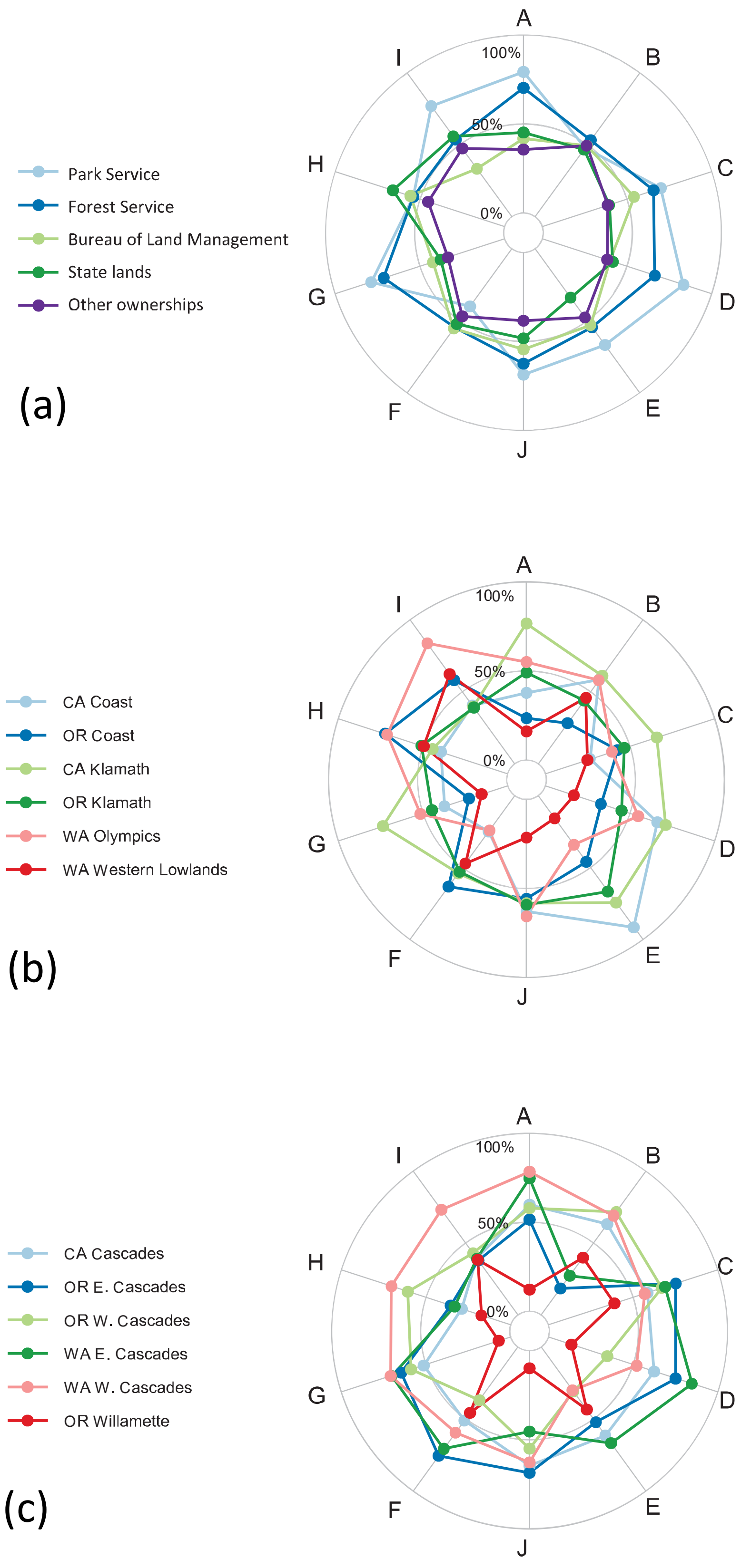

SI Table S1. Sources of data shown in Figures 1-6. Full references are provided at the end of Supplementary Information.

Metric	Source
Topodiversity	Carroll et al. (2017)
Outbound climatic velocity based refugia	Carroll et al. (2017)
Inbound climatic velocity based refugia	Carroll et al. (2017)
Shortest-path connectivity	Carroll et al. (2018)
Bird species refugia	Stralberg et al. (2018)
Tree species refugia	Stralberg et al. (2018)
Endemic species refugia	Carroll et al. (2010)
Composite priorities	Stralberg et al. (2020)
Aboveground carbon	Spawn et al. (2020)
Soil carbon (100 cm depth)	Hengl et al. (2017)
Land features used in analyses	
Yukon Territory planning regions	Yukon Land Use Planning Council (2018)
Peel Watershed planning units	Peel Watershed Planning Council (2011)
US land management agency ownerships	USGS (2018)
US national monuments	USGS (2018)
US inventoried roadless areas	USGS (2018)
US Conservation Reserve Program lands	U.S. Department of Agriculture (2004)
US conservation easements	National Conservation Easement Database (2020)
NFP physiographic provinces	USDA FS PNW Regional Ecosystem Office (2013)
NFP land use allocations	USDA FS PNW Regional Ecosystem Office (2013)

SI Text S1. Supplementary Methods.

Metrics of microrefugia potential

Climate refugia span a range of spatial scales (Morelli et al., 2020). Coarse-resolution metrics which identify macrorefugia (areas where broad-scale climate is relatively stable and suitable for persistence) can be complemented with other information that helps identify landscapes with greater potential to hold fine-scale microrefugia (small areas with locally favorable environments within otherwise unsuitable climates). Because microrefugia are often created by terrain-related factors, topographic diversity (topodiversity) data are useful for identifying areas where a heterogeneous physical environment (e.g., steep elevation gradients or diverse aspects) increases the likelihood that species will be able to find nearby suitable habitat as climate changes. Because they are mapped using topographic data rather than projections of future climate, these metrics are more robust to uncertainty concerning the pace of climate change. The topographic diversity metric used here, as presented in Carroll et al. (2017), represents the mean Euclidean environmental distance between all pairs of pixels within a spatial neighborhood of 27 by 27 km. (All data sources are listed in Table S1).

Climate velocity based refugia metrics

Macrorefugia can be mapped using a metric termed climate velocity, the speed that an organism needs to travel to keep pace with climate. Climatic macrorefugia are areas of low enough velocity that an organism can remain within their suitable climate tolerances even as climate change is changing. Climatic velocity is influenced by processes at several scales ranging from local topography to location in relationship to global climate circulation patterns. Metrics shown here were calculated as anomalies from the current (1981–2010) projected temperature and precipitation to the 2071–2100 period, based on an ensemble mean of 15 representative CMIP5 AOGCMs for representative concentration pathway (RCP) 8.5 (Wang et al., 2016).

Outbound or forward climatic velocity measures the distance between a site’s current climate type and the nearest site with the same climate type under future climates. This represents the rate at which an organism currently at a location must move to find future suitable climate. Outbound velocity, which provides information on the ability of resident species and ecosystems to persist regionally, will often be high in alpine areas because reaching

the nearest analogous future climate may require dispersal to distant mountaintops. A refugia metric, in which higher values represent areas with greater resilience to climate change, is derived by taking the logarithm of velocity values and then inverting them so that highest velocity areas have the lowest refugia score and vice versa.

Inbound or backward climate velocity measures the distance between a site's future climate type and the nearest site with the same climate type under current climates. This represents the rate at which organisms adapted to a location's future climate will need to move to colonize that location. Inbound velocity represents the distance and rate at which organisms adapted to a location's future climate will need to move to reach that location, and reflects a location's ability to serve as a refugium for species and ecosystems. Inbound velocity is generally low in alpine areas, because adapted organisms can reach the site from nearby downslope locations. Values are often high in valley bottom habitat because organisms must travel longer distances to colonize these locally new habitat conditions. Refugia analyses often focus primarily on areas with low inbound rather than outbound climatic velocity (Stralberg et al., 2018), but the two metrics are complementary.

Connectivity metrics

Although some organisms will be able to persist in place as climate changes, the persistence of many species will depend areas that facilitate dispersal to newly climatically suitable habitat. Climate connectivity areas or "climate corridors" are areas that form the best route between current climate types and where those climates will occur in the future under climate change. Climate connectivity areas are distinct from refugia and thus poorly captured by many existing conservation strategies. Because dispersing organisms may need to avoid hostile climates, these routes are often circuitous rather than the straight-line paths, as is assumed when measuring standard climatic velocity. Broad-scale topography and climate influence connectivity paths, and routes often funnel along north-south trending passes and valley systems and along the leeward or drier slopes of north-south trending mountain ranges. Human land use may further constrain the ability of species to disperse through these areas.

There are several types of climate connectivity metrics. The values used here, based on analyses presented in Carroll et al. (2018), represent the maximum of outbound and inbound shortest-path connectivity.

Climatic niche model based refugia metrics

Macrorefugia based on climate velocity represent a coarse-filter (non-species-specific) surrogate informing conservation of ecosystems and the vast majority of taxa for which detailed information is lacking. Ideally, however, such data should be complemented with fine-filter (species-specific) metrics focusing on individual species where that information is available. Macrorefugia for individual species can be mapped using a metric called biotic velocity that combines data from climate projections with data on the distributions of individual species. Climatic niche models based on correlations between species distributions and current climatic conditions are then projected forward to predict distribution under future climates.

Biotic velocity represents the distance between a site and the nearest site projected to be climatically suitable for the species under future projected climates. Biotic velocities provide a lower estimate of migration requirements than does climatic velocity because the metric assumes local populations can adapt to any climatic conditions found within the full range of the species' current distribution. When compared to refugia defined by climate types, biotic velocity highlights the influence of biogeographic factors (including past refugia locations) which have made certain regions more biodiverse than expected based on climate alone. Biotic-velocity-based refugia vary depending on the species considered. Stralberg et al. (2018) developed refugia metrics from biotic velocities for 268 songbird species and 324 tree species in the US and Canada.

Niche models for narrowly-distributed (rare and endemic) species is necessarily developed at regional rather than continental extents. Carroll et al. (2010) developed distribution models integrating climate data with vegetation variables for 131 localized species in the US Pacific Northwest, and then identified areas where current and future habitat for dispersal-limited species is in proximity, which we term here "endemic species refugia".

We also considered composite conservation values developed by Stralberg et al. (2020) from a systematic prioritization of conservation values across North America based on the

metrics described above representing velocity-based and species-based macrorefugia, topodiversity, and climate connectivity. Ecosystem-based climate mitigation potential was represented by a harmonized global map of above-ground biomass carbon density (Spawn et al. 2020) and a global map of predicted soil organic carbon (Hengl et al., 2017).

In order to scale the values of different metrics for comparison in starplots and maps, we scaled the metrics to 1000 equal area quantiles across the extent of interest (North America (or extent of datasets if smaller than North America) for Figs. 2, 3, 5, and 6, the Yukon Territory for Fig. 4, and the Northwest Forest Plan and surrounding region for Fig. 7).

REFERENCES

Carroll C, Dunk JR, Moilanen A (2010) Optimizing resiliency of reserve networks to climate change: multispecies conservation planning in the Pacific Northwest, USA. Global Change Biology, 16, 891-904.

Carroll C, Parks SA, Dobrowski SZ, Roberts DR (2018) Climatic, topographic, and anthropogenic factors determine connectivity between current and future climate analogs in North America. Global Change Biology, 24, 5318-5331.

Carroll C, Roberts DR, Michalak JL et al. (2017) Scale-dependent complementarity of climatic velocity and environmental diversity for identifying priority areas for conservation under climate change. Global Change Biology, 23, 4508-4520.

Hengl T, Mendes De Jesus J, Heuvelink GBM et al. (2017) SoilGrids250m: Global gridded soil information based on machine learning. PLoS One, 12, e0169748.

Morelli TL, Barrows CW, Ramirez AR et al. (2020) Climate-change refugia: biodiversity in the slow lane. Frontiers in Ecology and the Environment, 18, 228-234.

National Conservation Easement Database (2020). National Conservation Easement Database. www.conservationeasement.us.

Peel Watershed Planning Council (2011). Peel Watershed planning units. peel.planyukon.ca.

Spawn SA, Sullivan CC, Lark TJ, Gibbs HK (2020) Harmonized global maps of above and belowground biomass carbon density in the year 2010. Scientific Data, 7, 112.

Stralberg D, Carroll C, Pedlar JH, Wilsey CB, McKenney DW, Nielsen SE (2018) Macrorefugia for North American trees and songbirds: Climatic limiting factors and multi-scale topographic influences. Global Ecology and Biogeography, 27, 690-703.

Stralberg D, Carroll C, Nielsen SE (2020) Toward a climate-informed North American protected areas network: Incorporating climate-change refugia and corridors in conservation planning. Conservation Letters, 13, e12712.

U.S. Department of Agriculture (2004). U.S. Conservation Reserve Program (CRP) - Acreage by County. databasin.org/datasets/69826c5d16c24057ac590529f493913a.

USDA FS PNW Regional Ecosystem Office (2013). Land use allocations, 2013. www.fs.fed.us/r6/reo/library/maps.php.

USDA FS PNW Regional Ecosystem Office (2013). Physiographic Provinces, 2013. www.fs.fed.us/r6/reo/library/maps.php.

USGS (2018) Protected Areas Database of the United States (PAD-US), version 2.0. USGS Gap Analysis Program (GAP). Available at https://doi.org/10.5066/P955KPLE.

Yukon Land Use Planning Council (2018). Surveyed Cadastral Framework for Yukon First Nations and Tetlit Gwich'in settlement lands. planyukon.ca.

Wang T, Hamann A, Spittlehouse D, Carroll C (2016) Locally Downscaled and Spatially Customizable Climate Data for Historical and Future Periods for North America. PLoS One, 11, e0156720.
